# Quercetin induces HepG2 cell apoptosis by inhibiting fatty acid biosynthesis

**DOI:** 10.3892/ol.2014.2159

**Published:** 2014-05-19

**Authors:** PENG ZHAO, JUN-MIN MAO, SHU-YUN ZHANG, ZE-QUAN ZHOU, YANG TAN, YU ZHANG

**Affiliations:** 1 Health Management Center, Hangzhou Sanatorium of PLA, Hangzhou, Zhejiang 310007, P.R. China; 2 Department of Traditional Chinese Medicine, Hangzhou Sanatorium of PLA, Hangzhou, Zhejiang 310007, P.R. China; 3 Department of Clinical Laboratory, Hangzhou Sanatorium of PLA, Hangzhou, Zhejiang 310007, P.R. China

**Keywords:** quercetin, cancer, fatty acid synthase, HepG2 cells, apoptosis

## Abstract

Quercetin can inhibit the growth of cancer cells with the ability to act as a ‘chemopreventer’. Its cancer-preventive effect has been attributed to various mechanisms, including the induction of cell-cycle arrest and/or apoptosis, as well as its antioxidant functions. Quercetin can also reduce adipogenesis. Previous studies have shown that quercetin has potent inhibitory effects on animal fatty acid synthase (FASN). In the present study, activity of quercetin was evaluated in human liver cancer HepG2 cells. Intracellular FASN activity was calculated by measuring the absorption of NADPH via a spectrophotometer. MTT assay was used to test the cell viability, immunoblot analysis was performed to detect FASN expression levels and the apoptotic effect was detected by Hoechst 33258 staining. In the present study, it was found that quercetin could induce apoptosis in human liver cancer HepG2 cells with overexpression of FASN. This apoptosis was accompanied by the reduction of intracellular FASN activity and could be rescued by 25 or 50 μM exogenous palmitic acids, the final product of FASN-catalyzed synthesis. These results suggested that the apoptosis induced by quercetin was via the inhibition of FASN. These findings suggested that quercetin may be useful for preventing human liver cancer.

## Introduction

Fatty acid synthase (FASN), a metabolic enzyme that catalyzes the synthesis of long-chain fatty acids, is expressed at high levels in adipose tissues and a variety of human cancers, including liver, breast, prostate, endometrium, ovary, colon, lung and pancreatic cancer (1–10). Although the mechanism of FASN overexpression is unknown, it appears to be upregulated during the early stages of tumorigenesis (11). This differential expression between normal and neoplastic tissues makes FASN a potential diagnostic tumor marker (12).

Numerous studies suggest that obesity and excess weight play a prominent role in the incidence and progression of various types of cancer (13). Obesity has been associated with a higher risk and a poor prognosis of cancer in multiple studies (14–19). According to a previous study, obesity can increase the mortality of patients with cancer of the liver, breast and kidneys, among others (20). The potential of fatty acid synthesis as a target pathway for chemotherapy has been identified by studies with FASN inhibitors (21).

**Figure 1:**
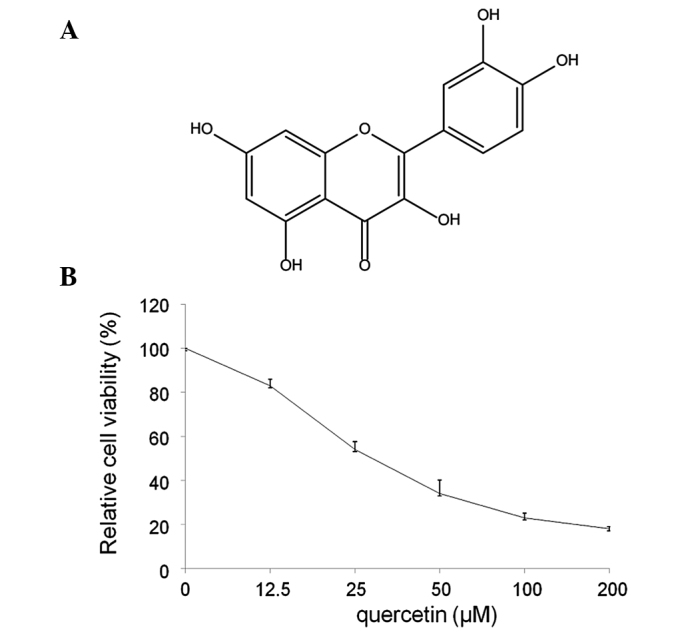
Dose-dependent inhibitory effects of quercetin on the viability of HepG2 cells. (A) Chemical structure of quercetin. (B) Cell viability was determined by MTT assay. HepG2 cells were incubated with quercetin for 24 h at the concentrations of 0–200 μM. IC_50_=24 μM. Bars represent the mean ± SD.

Studies suggest that dietary polyphenols, such as flavonoids, exert high inhibitory effects on FASN (22–28). Quercetin (3,3′,4′,5,7-pentahydroxyflavone) (Fig. 1A), an important dietary flavonoid present in red onions, apples, berries, citrus fruits, tea and red wine (29), exhibits antioxidant, anti-inflammatory, anti-obesity and anticancer properties (30). Quercetin has received increasing attention as a pro-apoptotic flavonoid with specific, and almost exclusive, effects on tumor cells rather than normal, non-transformed cells (31, 32).

Quercetin has been reported to provide an improved health status to its consumers, particularly with regard to obesity and diabetes (33). Studies have demonstrated that quercetin can modestly reduce weight and regulate the expression of genes related to *in vitro* adipogenesis (34,35). However, the mechanisms by which quercetin exerts these anticancer and anti-obesity effects remains unclear.

Therefore, the present study aimed to examine whether the anticancer activity of quercetin is associated with its anti-obesity effects. This study investigated the inhibitory effect of quercetin on human liver HepG2 cancer cells with overexpression of FASN.

## Materials and methods

### Reagents and antibodies

Quercetin, acetyl-CoA, alonyl-CoA, dexamethasone, Hoechst 33258, insulin, NADPH, MTT dye, 3-isobutyl-1-methylxanthine, palmitic acid, EDTA and DTT were purchased from Sigma-Aldrich (St. Louis, MO, USA). Dulbecco’s modified Eagle’s medium (DMEM) and fetal bovine serum were purchased from Gibco-BRL (Gaithersburg, MD, USA) and the penicillin-streptomycin was purchased from Oriental Bio-Technology Co., Ltd. (Beijing, China). Rabbit anti-human polyclonal FASN and mouse anti-human monoclonal GAPDH antibodies were purchased from Cell Signaling Technology, Inc. (Beverly, MA, USA).

### Cell culture

Human liver cancer HepG2 cells were obtained from the Cell Bank of the Chinese Academy of Sciences (Shanghai, China). Cells were incubated in DMEM (high-glucose), 10% fetal bovine serum and 100 U/ml penicillin-streptomycin.

### MTT assay

HepG2 cells were seeded in a 96-well plate (5×10^3^ cells/well) and then treated with quercetin at different concentrations for 24 h. Thereafter, 20 ml of MTT solution [5 mg MTT/ml in phosphate-buffered saline (PBS)] was added into each well of a microtiter plate and incubated for 4 h at 37°C. The resultant formazan product was dissolved in 200 ml dimethylsulfoxide/well, and its concentration was measured at 492 nm by a microplate reader (Model EL 307C; BioTek, Shanghai, China).

### Cell lysis and immunoblotting

Cells were lysed as previously described (36) and the cell lysates were heated in a water bath to fully denature the proteins. The proteins were then separated by SDS-PAGE [Bio-Rad Laboratories (Shanghai) Ltd., Shanghai, China] and transferred to polyvinylidene difluoride membranes (Immobilon; Millipore, Billerica, MA, USA). Immunoblotting was performed with antibodies against FASN and GAPDH, and visualized using an enhanced chemiluminescence light detection kit (Amersham, Piscataway, NJ, USA).

### Cell apoptosis assay

HepG2 cells were seeded in 12-well culture dishes (5×10^4^ cells/well). Following experimental treatment with 25 and 50 μM quercetin for 24 h, cells were washed twice with PBS, stained with Hoechst 33258 (5 mg/ml) for 5 min in the dark, and then washed extensively three times with PBS. Nuclear staining was examined under a fluorescence microscope (Nikon LH-M100CB; Jirui Co., Ltd., Suzhou, China) and images were captured using Image-Pro Plus software (MediaCybernetics, Silver Spring, MD, USA).

### Intracellular fatty acids assay

The amount of intracellular fatty acid was determined by the Fatty Acid Assay kit (Lab-Bio Co., Ltd., Beijing, China). Briefly, HepG2 cells were seeded in 100-mm cell culture dishes. Following experimental treatment, cells were washed twice with PBS and then extracted by homogenization with 200 μl chloroform-Triton X-100 (1% Triton X-100 in pure chloroform; Shanghai XiTang Biotechnology Co., Ltd., Shanghai, China) in a microhomogenizer. Subsequently, the extract was centrifuged for 5–10 min at high speed (16,000 × g). The organic (lower) phase was collected and air-dried at 50°C to remove the chloroform, followed by vacuum-drying for 30 min to remove trace chloroform. The dried lipids were dissolved in 200 μl Fatty Acid Assay buffer by vortexing extensively for 5 min. Next, 2 μl acyl-CoA synthetase reagent was added to all sample wells and the samples were incubated at 37°C for 30 min. Following this, 50 μl reaction mix containing 44 μl Fatty Acid Assay buffer, 2 μl Fatty Acid Assay probe, 2 μl enzyme mix and 2 μl enhancer, was added to the test samples. The samples were then incubated for 30 min at 37°C, whilst being protected from light. The colorimetric assay was conducted by measuring the absorbance at 570 nm using a microplate reader.

### Cell FASN activity assay

FASN activity in cells was assessed as described previously (37). Briefly, cells were harvested, pelleted by centrifugation at 18,000 × g for 30 min, resuspended in cold assay buffer (100 mM potassium phosphate buffer, 1 mM EDTA, 0.6 mM PMSF and 1 mM dithiolthreitol, pH 7.0) ultrasonically disrupted and centrifuged at 16,000 × g for 30 min at 4°C. The supernatant was then collected for the overall reaction assay. A total of 25 ml supernatant was added to the reaction mix containing 25 mM KH_2_PO_4_-K_2_HPO_4_ buffer, 0.25 mM EDTA, 0.25 mM dithiothreitol, 30 mM acetyl-CoA, 100 mM malonyl-CoA and 350 mM NADPH (pH 7.0), in a total volume of 200 ml. Protein content in the supernatant was determined using a bicinchoninic acid assay (Pierce, Rockford, IL, USA) and results were expressed as the specific activity of FASN at the same protein concentration as the control group (0 μM quercetin).

### Palmitic acid assay

HepG2 cells were exposed for 24 h to various concentrations of quercetin (0, 25 and 50 μM) in the presence of exogenous palmitic acid (0, 25 and 50 μM), the end product of the FASN reaction. Next, the relative cell viabilities were analyzed by MTT assay.

### Statistical analysis

The results were analyzed by one way analysis of variance (origin 8.0). P<0.05 was considered to indicate a statistically significant difference, while P<0.01 was considered to indicate a markedly significant difference.

## Results

### Inhibitory effects of quercetin on viability of HepG2 cells in vitro

To identify whether quercetin influences the survival of HepG2 cells, cells were treated with 0–200 μM quercetin and cell viability was examined by MTT assay. As shown in Fig. 1B, HepG2 cell viability was reduced to 52% with 25 μM quercetin and to 34% with 50 μM quercetin. Cell growth was markedly suppressed by 82% following treatment with 200 μM quercetin, when compared with the control (0 μM). Quercetin showed high inhibition of cell population growth in a dose-dependent manner with a 50% growth inhibitory concentration (IC_50_) value of 24 μM.

### Quercetin inhibits FASN expression and activity in HepG2 cells

**Figure 2:**
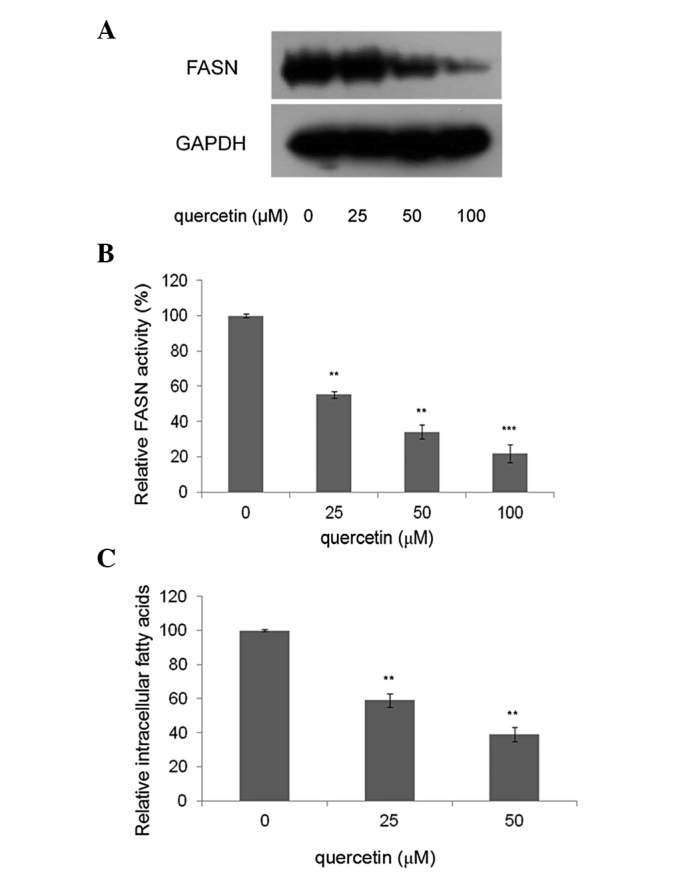
Effects of quercetin on FASN expression and activity. (A) Effect of quercetin on FASN expression. Cells were treated with 0, 25, 50 and 100 μM quercetin. After 24 h, cells were harvested and analyzed by western blotting. (B) FASN activity assay was performed as described in Materials and methods. Data were normalized to those of control cells without quercetin (0 μM). Relative FASN activity is presented as the mean ± SD. ^*^P<0.05, ^**^P<0.01 and ^***^P<0.001, compared with the control, respectively. (C) HepG2 cells were treated with quercetin at various concentrations (0, 25 and 50 μM) for 24 h. The amount of intracellular fatty acid was then determined by the Fatty Acid Assay kit. Data were expressed as the mean ± SD. (n=3). ^**^P<0.01, compared with the respective control. FASN, fatty acid synthase

The effect of quercetin on the expression of FASN in HepG2 cells. was investigated. As shown in Fig. 2A, compared with the control, the cells treated with quercetin showed markedly lower levels of FASN. This suggests that the FASN expression levels were significantly suppressed by quercetin. Compared with the control, quercetin significantly inhibited the intracellular FAS activity in a dose-dependent manner. As shown in Fig. 2B, HepG2 cells were treated with quercetin at a concentration of 25, 50 and 100 μM for 24 h. Intracellular FASN activity was reduced to 55.6, 34.3 and 22.1%, respectively, compared with control.

### Quercetin reduces intracellular fatty acids in HepG2 cells

The levels of intracellular fatty acids in HepG2 cells treated with 25 and 50 μM quercetin were measured, as these concentrations were able to reduce cell viability with IC_50_ values of 25 and 50 μM and downregulate FASN expression significantly. The results showed that the levels of intracellular fatty acids in treated cells decreased by 40.6 and 60.8%, compared with the control (0 μM quercetin) (Fig. 2C).

### Quercetin induces HepG2 cells apoptosis

**Figure 3:**
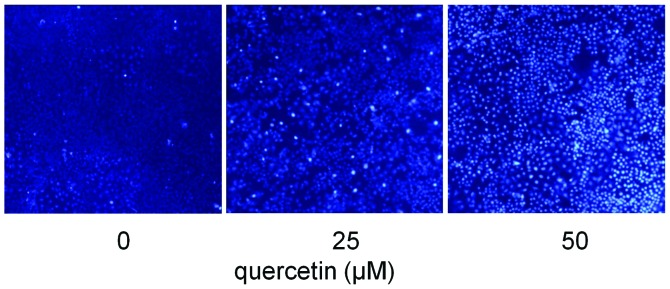
Apoptotic effect of quercetin on HepG2 cells. Cell culture was performed as described in Materials and methods. Photographs of HepG2 cells were taken after Hoechst 33258 staining. The concentrations of quercetin were 0, 25 and 50 μM.

In order to examine whether the inhibitory effect of quercetin on HepG2 cells was due to apoptotic cell death, apoptotic events of Hoechst 33258 staining were investigated. Following exposure to three different concentrations of quercetin (0, 25 and 50 μM) for 24 h, apoptosis of HepG2 cells was demonstrated by Hoechst 33258 staining, revealing cell membrane permeability increases and nuclear condensation in a dose-dependent manner (Fig. 3).

### Palmitic acid rescues cell apoptosis induced by quercetin

**Figure 4:**
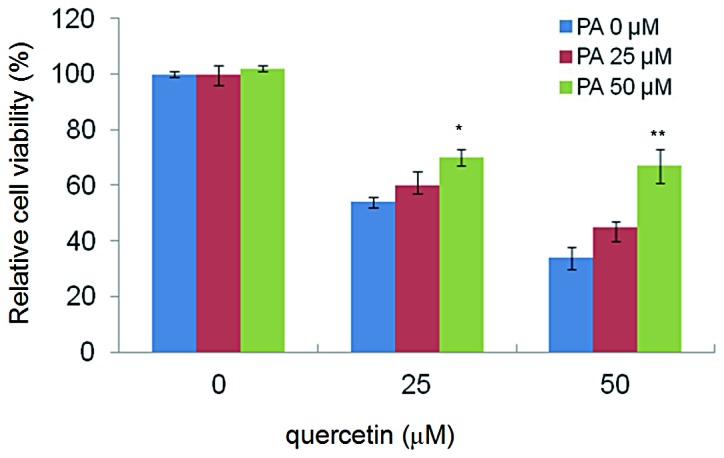
Sodium palmitate rescued HepG2 cell apoptosis induced by quercetin. Sodium palmitate (0, 25 and 50 μM) was added to cells in the presence of 0, 25 and 50 μM quercetin. After 24 h, MTT assay was used to analyze the cell viability. Bars represent the mean ± SD.

To confirm that the cell apoptosis induced by quercetin was related to FASN inhibition, HepG2 cells were exposed to different concentrations of quercetin (0, 25 and 50 μM) for 24 h, in the presence of exogenous palmitic acid (0, 25 and 50 μM), the end product of the FASN reaction. Palmitic acid reduced the cytotoxic effects of quercetin, and the cell viabilities were restored significantly and in a dose-dependent manner (Fig. 4).

## Discussion

Dietary phytochemicals consist of a wide variety of biologically active compounds that are ubiquitous in plants, a number of which have been reported to have antitumor properties. Among these, quercetin, which is abundant in red onions, apples, berries, citrus fruits, tea and red wine, has been reported to have therapeutic potential for treating numerous types of human cancer (38–43). Quercetin is well-known for its benefits for weight control and cancer prevention. However, to date, no association has been reported between its anti-obesity and cancer prevention activities.

Inhibition of FASN in cancer cells has been found to induce apoptosis, which suggests that inhibiting intracellular FASN should be a reasonable way for the treatment of cancer (44,45). Li and Tian have reported that quercetin is a natural and potent FASN inhibitor with an IC_50_ value of 4.29±0.34 μM (46). The present study showed that quercetin induced liver cancer cell apoptosis via inhibition of FASN.

FASN is a key enzyme participating in lipogenesis and the *de novo* synthesis of palmitate from Ac-CoA, Mal-CoA and NADPH, and plays an important role in converting excess carbon intake into fatty acids for energy storage (2,47). In normal tissue, FASN levels are generally low, as the requirement of quiescent cells for fatty acids is generally provided via dietary fatty acids. However, in rapidly proliferating cancer cells, such as liver, prostate, ovarian, breast, endometrial and thyroid carcinomas, FASN is overexpressed (2). Overexpression of FASN in cancer cells suggests that tumors require higher levels of fatty acids than can be acquired from the circulation, but also indicates higher levels of endogenous production. Elevated expression of FASN has been linked to poor prognosis and reduced disease-free survival in numerous types of cancer (48). RNAi knockdown experiments have shown that multiple cancer cell lines depend on FASN for proliferation and survival. FASN appears to play a key role in tumor initiation and propagation for a number of malignancies, and represents an attractive target for cancer treatment. Although the ultimate mechanism of cancer-associated FASN overexpression is not completely understood, it has been shown that FASN inhibitors such as C75 and orlistat are promising potential anticancer drugs. It is necessary to discover additional FASN inhibitors that may be applied practically in the treatment of cancer.

High expression of FASN in human liver, breast, colorectal, prostate, endometrial, ovary and thyroid cancer supports the hypothesis that FASN is essential for generating cell membranes during tumor cell proliferation (49). In the present study, it was found that quercetin not only exerted a high inhibitory effect on intracellular FASN, but also influenced the normal life cycle of cancer cells (Fig. 1B and C). These results suggested that FASN, a target for treating cancer, was also a target of quercetin.

The activity of FASN in cells affects the levels of intracellular fatty acids, as FASN plays a key role in *de novo* fatty acid biosynthesis. Considering that quercetin has been found in numerous edible plants, it may be safe to assume that a high intake of quercetin is safe.

In the current study, similar to reported FASN inhibitors, such as C75 and cerulenin (21), quercetin could induce apoptosis in cancer cells (Fig. 2C). Previous studies have suggested that the mechanism of apoptosis through inhibiting FASN could be explained by the accumulation of malonyl-CoA, which was likely to trigger cancer cell death and induce apoptosis (50,51). It was proposed that certain signaling pathways involved in cell apoptosis were closely associated with the inhibition of FASN, which may help to explain why FASN inhibitors may potentially be used to treat cancer.

Certain studies, however, have shown that palmitic acid, the final product of FASN, is important for the formation of cell membranes (52). Therefore, the reduction of synthesized palmitic acid may be another reason to explain why the inhibition of FASN could induce apoptosis. In the current study, it was found that the reduced cell viabilities induced by quercetin treatment could be rescued by adding exogenous palmitic acid, which provided strong evidence for the cell membrane thesis (Fig. 2D and Fig. 3C).

In conclusion, the present study demonstrated that quercetin could induce HepG2 cells apoptosis via inhibition of intracellular FASN activity and downregulation of FASN expression. The finding that palmitic acid rescued quercetin-induced apoptosis in cancer cells confirmed that the induction of apoptosis was associated with the inhibition of FASN. As quercetin showed potent inhibitory effects on the proliferation of HepG2 cells, it has the potential to be developed into a candidate drug for treating human liver cancer.
